# Bundles reduce anastomosis leak in patients undergoing elective colorectal surgery. A propensity score-matched study

**DOI:** 10.3389/fsurg.2023.1119236

**Published:** 2023-02-27

**Authors:** M Baeza-Murcia, G Valero-Navarro, E Pellicer-Franco, V Soria-Aledo, M Mengual-Ballester, J. A Garcia-Marin, L Betoret-Benavente, J. L Aguayo-Albasini

**Affiliations:** ^1^ Servicio de Cirugía General y Digestiva, Hospital General Universitario Morales Meseguer, Murcia, Spain; ^2^ Grupo de Investigación Quirurgica en Area de Salud, Instituto Murciano de Investigación Biosanitaria Pascual Parrilla, Murcia, Spain

**Keywords:** bundle, anastomosis leakage, colorectal surgery complicatios, bowel mechanical preparation, inflammatory marker

## Abstract

**Background:**

anastomosis leak still being a handicap in colorectal surgery. Bowel mechanical preparation and oral antibiotics are not a practice recommended in many clinical practice guides. The aim is to analyse the decrease in frequency and severity of postoperative complications, mainly related to anastomotic leak, after the establishment of a bundle.

**Methods:**

Single-center, before-after study. A bundle was implemented to reduce anastomotic leaks and their consequences. The Bundle group were matched to Pre-bundle group by propensity score matching. Mechanical bowel preparation, oral and intravenous antibiotics, inflammatory markers measure and early diagnosis algorithm were included at the bundle.

**Results:**

The bundle group shown fewer complications, especially in Clavien Dindós Grade IV complications (2.3% vs. 6.2% *p* < 0.01), as well as a lower rate of anastomotic leakage (15.5% vs. 2.2% *p* < 0.01). A significant decrease in reinterventions, less intensive unit care admissions, a shorter hospital stay and fewer readmissions were also observed. In multivariate analysis, the application of a bundle was an anastomotic leakage protective factor (OR 0.121, *p* > 0.05)

**Conclusions:**

The implementation of our bundle in colorectal surgery which include oral antibiotics, mechanical bowel preparation and inflammatory markers, significantly reduces morbidity adjusted to severity of complications, the anastomotic leakage rate, hospital stay and readmissions.

**Register study:**

The study has been registered at clinicaltrials.gov Code: nct04632446.

## Introduction

The safety of patients undergoing colorectal surgery has significantly improved during the past 50 years due to the progress in preoperative preparation, surgical technique and postoperative treatment. Even so, there are still postsurgical complications, with a current morbidity of close to 40% in elective surgery (1).

Among the complications of colorectal surgery, Surgical Site Infection (SSI) is the most important one, reaching up 20% (2) and represents the highest rates in all major abdominal surgery. This is probably due to the influence of Organ-Space Infection, which includes anastomosis leak (AL) and whose origin seems to differ from Incisional SSI. Organ-space SSI alone accounts for 23% of re-hospitalizations, 60% of reoperations and 29% of admissions to Intensive Care Units (ICU), trebling hospital stay (3). The incidence of AL varies between authors, from 2 to 14% in colon surgery and from 2 to 29% in rectal surgery (4).

Due to the frequency and severity of SSI in elective colorectal surgery, specific guidelines have been prepared in order to reduce this type of complications by using bundles or a series of measures aimed at improving postoperative results. Today, there is not just one bundle, but different groups (5–7) and societies (8) who have implemented different measures succeeding in significantly reducing SSI. Mechanical bowel preparation (MBP) and oral antibiotic prophylaxis have been two of the most frequently used measures. Although there is a broad consensus that antibiotic prophylaxis is essential before colorectal surgery, there is still controversy about whether antibiotics should be administered intravenously, orally, or combined. On the other hand, the role of MBP alone or with oral antibiotics has been extensively discussed.

The purpose of this study is to evaluate the improved frequency and severity of complications and the morbidity associated with anastomosis leak after de use of a bundle in patients undergoing elective colorectal surgery.

## Methods

We conducted a study before and after implementing a bundle with 5 new measures. The Pre-bundle cohort consisted of 95 consecutive patients undergoing elective colorectal surgery with anastomosis, from October 1, 2017 to May 30, 2018. The incidence of complications of these patients was recorded and their C-Reactive Protein (CRP) reference levels were obtain as a marker for early diagnosis of anastomotic dehiscence by applying the ROC curves and calculating the pathological reference value using the Youden's index (>15 mg/dl on the third postoperative day, >10 mg/dl on the fourth postoperative day and >9 mg/dl on the fifth postoperative day) (9). These values were used in the bundle for early diagnosis of complications.

The sample size of the patients in the Bundle group was calculated for a decrease in serious complications (grades IV and V of the Clavien-Dindo Classification) to 6%, having the Pre-bundle group as a reference.

The inclusion criteria were: patients over 18 years old, signed the informed consent and underwent elective colorectal surgery due to malignant or benign neoplasia with anastomosis during surgery. Patients who required transfer to another center or those with fatal evolution (death) before the third postoperative day, were excluded from the study. Finally, the Bundle group consisted of 139 patients.

**Figure 1:**
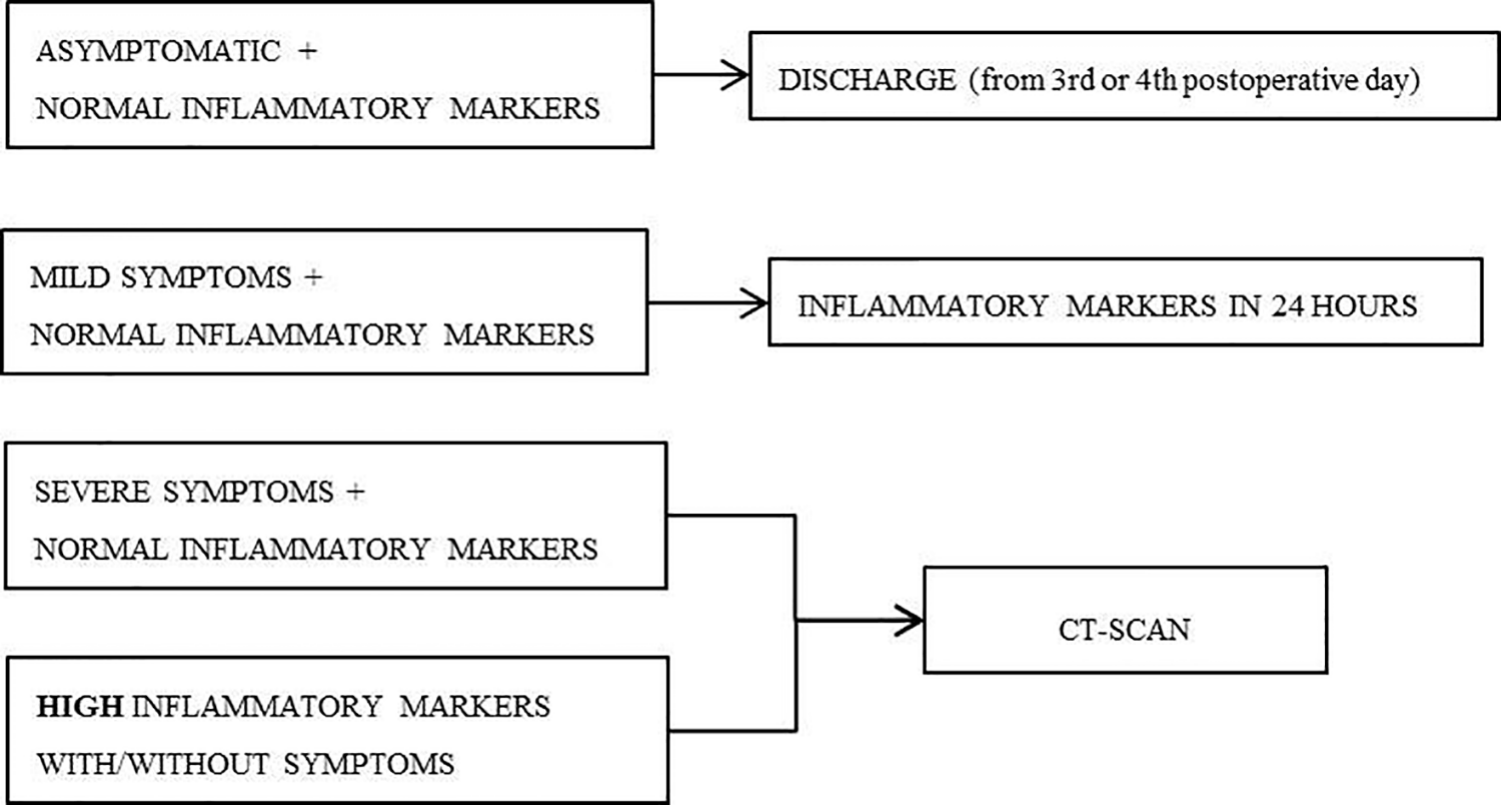
Algorithm for the early diagnosis of anastomotic leak.

The bundle consisted on preoperative and postoperative measures (Table 1) and included an algorithm for the early diagnosis of anastomosis leak (Figure 1). All our patients are assessed daily by a coloproctology unit surgeon, since the first postoperative day, and a CRP were measured ant 3rd, 4th and 5th postoperative day.

**Table 1:** Measures implemented in the bundle*.*

Preoperative measures	Postoperative measures
Mechanic bowel preparation	**Regular blood tests on the 3rd, 4th and 5th postoperative day** (hemogram, venous blood gas, biochemical profile and CPR)
Oral antibiotic prophylaxis (Neomycin 1 gram and Metronidazole 1 gram in 3 doses on the day prior surgery, at 13, 14 and 23 h)	**Implementation of the algorithm for early diagnosis of AL**
Single dose intravenous antibiotic therapy (Cefminox 2 grams)	

The asymptomatic patients with CRP below the calculated cut-off point were discharged on the third or fourth postoperative day. Patients with mild symptoms (such as feeding intolerance, absence of intestinal transit or abdominal discomfort) and markers within normal ranges, had another blood test performed after 24 h. Patients with serious symptoms (like fever, hemodynamic instability or peritoneal irritation signs) and normal markers had an abdominal and pelvic Computed Tomography scan (CT-scan) with double or triple contrast performed. Patients with high inflammatory markers had a CT-scan performed, whether they had symptoms or not. All the patients of the Pre-Bundle group received IV Cefminox 2gr within the hour prior to surgery and oral MBP, with sodium picosulfate.

The study was single-center. Patient selection, data collection, and later follow-up were conducted prospectively during the first 30 days after surgery. The preoperative, intraoperative and postoperative variables of all patients were collected.

**Figure 2:**
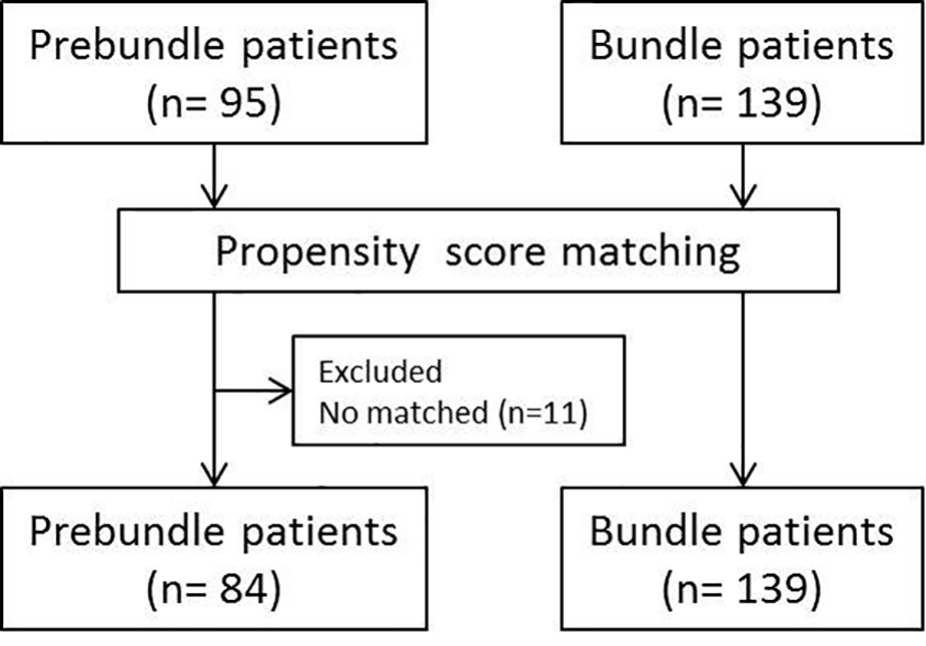
Study population and flowchart showing patient group before and after propensity score matching.

Data processing and statistical analysis were performed using SPSS 24.0. In order to obtain two comparable homogeneous groups, a propensity score matching analysis was performed. Confounding variables used to set the propensity score were age, sex, Charlson index, American Society of Anaesthesiologists (ASA) classification, preoperative steroids and surgical approach. On the basis of multi-factor logistic analysis, the propensity score was calculated with a caliper width of 0.2, obtaining two groups with 84 patients in the Pre-bundle Group and 139 in the Bundle group (Figure 2). At first, a univariate analysis was performed to compare the groups by using chi-squared distribution for discrete variables (considering standardized residual in tables bigger than 2 × 2); and Students' t-distribution for continuous variables (using Levene's test to assess the distribution of variances). For the purpose to avoid any bias, a subgroup analysis was performed according the colon condition (benign or malignant).

In order to assess the possible prognostic factors of the severity of morbidity, ICU stay and presence of anastomosis leak, a multivariate analysis was conducted using backward stepwise logistic regression to describe the significant variables for our study.

The study has been approved by the appropriate institutional ethics committee and have been performed in accordance with the ethical standards as laid down in the 1,964 Declaration of Helsinki and its later amendments or comparable ethical standards. Patients have consented to participate and to publication.

## Results

The 84 patients of the Pre-bundle group were compared with a cohort of 139 patients subjected to the bundle (Bundle group), operated between March 2019 and May 2020. Adherence to the bundle was 99.3% for mechanic preparation and 95.7% for oral antibiotic prophylaxis. However, the adherence to the implementation of the diagnostic algorithm for postoperative complications was 87%.

Both groups were homogeneous (sex, age and BMI, among others), although the Bundle group presented with higher Charlson Index (Table 2). Both were also homogeneous regarding surgical approach, procedure, surgical team, ostomy confection, metastasis, carcinomatosis and Possum score (Table 3).

**Table 2:** Univariate descriptive analysis of the preoperative variables comparing both groups*.*

		Pre-bundle	Bundle	*p*
Total patients		84	139	N/A
Sex	Male	56 (66.7%)	86 (61.9%)	0.471
	Female	28 (33,3%)	53 (38.1%)	
Age (years)		64,65 (±13.8)	64,51 (±14.6)	0.940
Charlson Index		**3.06 (±1.9)**	**4.43** (**±2.7)**	**0.001**
BMI (kg/m2)		27.25 (±5.0)	27.66 (±4.6)	0.538
Albumin levels (g/dl)		4.22 (±0.48)	4.26 (±0.56)	0.608
ASA	I	9 (10.7%)	5 (3.6%)	0,102
	II	50 (59.5%)	82 (59%)	
	III	24 (28.6%)	46 (33.1%)	
	IV	1 (1.2%)	6 (4.3%)	
Smoking		15 (17.9%)	38 (27.3%)	0.107
Corticotherapy		1 (1,2%)	2 (1.4%)	0.876
Neoadjuvant therapy		4 (5.3%)	6 (5.6%)	0.966
Diagnosis	Colon neoplasia	59 (62.4%)	85 (61.2%)	0.128
	Rectal neoplasia	16 (19%)	22 (15.8%)	
	Sigmoid volvulus	0	2 (1.4%)	
	Reconstruction after Hartmann procedure	1 (1.2%)	7 (5%)	
	FAP	3 (3.2%)	0	
	Diverticular disease	4 (4.8%)	18 (12.9%)	
	Inflammatory disease	1 (1.2%)	5 (3.7%)	
Stage (T)	Tis-T2	33 (43.4%)	58 (54.2%)	0.303
	T3	35 (46.1%)	42 (39.3%)	
	T4	8 (10.5%)	7 (6.5%)	

N, number of patients; *p*, statistical significance; N/A, not applicable; ASA, American society of anesthesiologists; FAP, familial adenomatous polyposis; T, tumor size according to TNM staging for colorectal cancer; Tis, carcinoma *in situ*.

**Table 3:** Comparative analysis of the intraoperative variables.

		Pre-bundle	Bundle	*p*
Approach	Open surgery	44 (52.4%)	57 (41%)	0.098
	Laparoscopy	40 (47.6%)	82 (59%)	
Procedure	Right hemicolectomy	36 (42.9%)	55 (39.6%)	0.301
	Left hemicolectomy /Sigmoidectomy	26 (31%)	49 (35.4%)	
	Low anterior resection	17 (20.2%)	22 (15.8%)	
	Subtotal colectomy	4 (4.8%)	3 (2.2%)	
	Bowel transit reconstruction	1 (1.2%)	8 (5.8%)	
	Segmental resection	0 (0%)	2 (1.2%)	
POSSUM score		10.9 (±1.4)	10.95 (±1.2)	0.176
Surgeon	Colorectal	71 (84.5%)	123 (88.5%)	0.394
	General	13 (15.5%)	16 (11.5%)	
Protective stoma		15 (17.9%)	18 (12.9%)	0.317
Carcinomatosis		1 (1.2%)	0 (0%)	0.197
Liver metastasis		6 (7.1%)	6 (4.3%)	0.365

N, number of patients; p, statistical significance.

The percentage of patients who presented with complications in the Bundle group was lower than in the Pre-bundle group (34.5% vs. 46.4%), without reaching statistical significance, but when analyzing the distribution of the complications according to their gravity, we observed that the Bundle group mainly presented with mild complications, grade I of Clavien-Dindo classification (77% vs. 20.5% *p* = 0.001), whereas in the Pre-bundle group the rate of severe complications, grade IV of Clavien-Dindo classification, was significantly higher (23% vs. 6.2%, *p* = 0.001). Moreover, the incidences of Organ-space SSI (and therefore of AL), acute respiratory failure and dynamic ileus were lower in the Bundle group, *p* < 0.05. Mortality was similar in both series (2.4% in the Pre-bundle group vs. 2.1% in the Bundle group) (Table 4).

**Table 4:** Comparative analysis of postoperative complications.

		Pre-bundle	Bundle	*p*
Overall morbidity		39 (46.4%)	48 (34.5%)	0.07
Mortality		2 (2.4%)	3 (2.1%)	0.635
Clavien-Dindo^a^	I	8 (20.5%)	37 (77%)	**0**.001*
	II	17 (43,6%)	3 (6.2%)	
	III	3 (7.7%)	2 (4.1%)	
	IV	9 (23%)	3 (6.2%)	
	V	2 (5.1%)	3 (6.2%)	
Type of complication	Surgical wound infection (superficial and deep SSI)	2 (2.4%)	7 (5%)	0.362
	Organ-space SSI	14 (16.7%)	5 (3.6%)	**0**.001*
	Anastomotic dehiscence	13 (15.5%)	3 (2.2%)	**0**.001*
	Hemoperitoneum	6 (7.1%)	3 (2.2%)	0.067
	Lower gastrointestinal bleeding	5 (6%)	5 (3.6%)	0.536
	Intestinal ischemia	1 (1.2%)	0 (0%)	0.197
	Acute renal failure	6 (7.1%)	6 (4.3%)	0.365
	Acute respiratory failure	7 (8.3%)	3 (2.2%)	**0**.031*
	Febrile illness	15 (17.9%)	22 (15.8%)	**0**.**693**
	Postoperative adynamic ileum	27 (32.1%)	22 (15.8%)	**0**.004*
	Phlebitis	1 (1%)	9 (6.5%)	0.001*

N, number of patients, P, statistical significance, SSI, surgical site infection.

^a^ Calculated based on patients with complications.

Hospital stay for the Bundle group was shorter (6.3 vs. 11.4 days, *p* = 0.001), as well as the need for ICU (4.3% vs. 15.5%, *p* = 0.004), the re-hospitalization rate (4.3% vs. 13.1%, *p* = 0.017) and the need for imaging tests during the postoperative period, despite complying with the diagnostic algorithm (15.1% vs. 29.8%, *p* = 0.009), were significantly lower (Table 5).

**Table 5:** Comparative analysis of reoperations, re-hospitalizations, ICU management and stay.

	Pre-bundle	Bundle	*p*
Reoperation	11 (13.1%)	5 (3.6%)	**0**.008*
CT-scan after surgery	25 (29.8%)	21 (15.1%)	**0**.009*
ICU management	13 (15.5%)	6 (4.3%)	**0**.004*
Re-hospitalization	11 (13.1%)	6 (4.3%)	**0**.017*
Hospital stay	11.4 (±10.42)	6.3 (±4.17)	**0**.001*

N, number of patients; p, statistical significance; CT-scan, computed tomography scan; ICU, intensive care unit.

On the other hand, the laparoscopic approach was associated with less incidence of complications (18.8% vs. 47.5%, *p* = 0.001) and of severe complications (grades IV and V of Clavien-Dindo classification) than open surgery (4.1% vs. 11.9%, *p* = 0.001). However, these differences were not significant regarding AL, whose incidence was similar in both approaches (Table 6).

**Table 6:** Comparative analysis of the approach in relation to the postoperative complications and their seriousness.

		Open surgery	Laparoscopy	*p*
No complications		**45** **(44.6%)**	**91** **(74.6%)**	0.001*
Complications other than AL		**48** **(47.5%)**	**23** **(18.8%)**	
AL		8 (7.9%)	8 (6.6%)	
Seriousness of complications (Clavien-Dindo)	Grades 0-III	**89** **(88.2%)**	**117** **(95.9%)**	0.001*
	Grades IV–V	**12** **(11.9%)**	**5** **(4.1%)**	

p, statistical significance; AL, Anastomosis leak.

When analyzing the variables of the subgroups according to the condition (benign vs. malignant), we have obtained that both are also homogeneous with no differences in the preoperative and operative variables. Moreover, the morbidity, mortality, all complications and postoperative results were similar to the overall series both in benign and malignant subgroups.

The multivariate analysis included the variables related to AL occurrence: sex, age, ASA, Charlson Index, BMI, surgical approach and bundle implementation. The bundle itself was a protective factor for AL occurrence [OR 0.121,—CI 95% (0.033–0.446)]. Moreover, male sex was associated with a significantly higher risk of AL (OR 9.350, CI 95% 1.190–73.488).

## Discussion

Due to the high risk and repercussion of SSI and AL in colorectal surgery, many have been the strategies used throughout history to try to reduce them. In 1934, Poth (10) concluded that MBP on its own did not succeed in reducing the bacterial content in the colon; therefore, oral non-absorbable antibiotics were introduced (11, 12). Later on, with the detection of anaerobic microorganisms in the colon (13), an anaerobicidal agent, such as metronidazole, was added to neomycin, which, in combination with MBP, succeeded in reducing aerobic and anaerobic bacteria outgrowth in the sample (14), and reduced the incidence of SSI and AL (15), thus consolidating the principles of bowel preparation.

This trend has continued in the United States and Canada since the 80s (5–7, 16–31) with good results regarding SSI decrease. But this is not the case in Europe (32–36), where the ERAS® program (37) and the guidelines of the British National Institution of Health and Clinical Excellence 2008 (38) reject MBP and advocate the superiority of intravenous prophylaxis for SSI prevention, reporting an increased incidence of pseudomembranous colitis and antibiotic resistance associated with oral prophylaxis (39).

Due to the high morbidity resulting from the AL and the disparity of the results of the published works regarding how to avoid it, we decided to monitor the complication rate in our unit, which resulted in an incidence of infection of the surgical wound (superficial and deep) of 2.4% and an AL rate of 15.5%. Not only the overall incidence of complications but also their grade of severity was high, with 23% of grade IV complications according to Clavien-Dindo classification and 5.1% of grade V. Moreover, the mean hospital stay was 11 days with 13.1% of re-hospitalizations. After being aware of these figures, we created a bundle that allowed for decreasing the incidence of such complications and reducing their severity and repercussion on the patient.

With the implementation of the new bundle, we obtained a decrease in morbidity from 46.4% to 34.5%, although without reaching significant values. However, the severity of complications did change considerably in both groups. Most of the complications in the Bundle group were grade I of Clavien-Dindo classification (77% vs. 20.5% in the Pre-bundle group), and grade IV complications of Clavien-Dindo classification were significantly higher in the Pre-bundle group (23% vs. 6.2% in the Bundle group). Therefore, we can say that the implementation of the new measures drastically reduced the severe complications of elective colorectal surgery. The most relevant difference was the incidence of organ-space infection (16.7% to 3.6%) and particularly the incidence of anastomosis leak, which significantly decreased from 15.5% to 2.2% in the Bundle group (*p* = 0.001).

Other authors have published similar results on the decrease of SSI after the implementation of bundles. Lutfiya et al. (5) who, after implementing the measures of the American College of Surgeons “ACS NSQIP” (8), obtained a decrease in overall SSI at the expense of superficial and deep incisional infection (21.15% to 6.67%, *p* = 0.001). Weiser et al. (40) in 2018 conducted a study before and after the implementation of a bundle, in which they divided the patients according to their risk of SSI. The incidence of SSI decreased from 11% to 4.1% at the expense of the groups with intermediate or high risk of SSI. These differences were significant in the superficial and deep incisional infections. A much smaller range of measures than “ACS NSQIP” (8) was implemented in our study, thus facilitating compliance (7).

Studies such as Gorgun et al. (22) also found a decrease in overall SSI when implementing their bundle (11.8% to 6.6%, *p* = 0.001), associated with decreased organ-space infection (5.5% vs. 1.7%, *p* = 0.001). Likewise, Mulder et al. (24) also succeeded in significantly reducing overall SSI and AL, thus reducing hospital stay from 8 to 7 days. Like in our study, a laparoscopic approach was most frequently used in the group after the bundle implementation. In this line, we also observed that a laparoscopic approach yielded a lower complication rate, particularly severe complications (grade IV and V of Clavien-Dindo classification), than an open approach (4.1%vs.11.9%). It is worth noting that Mulder et al. administered oral antibiotic prophylaxis and intravenous prophylaxis, without mechanical bowel preparation. In our study, we opted for a combination of antibiotics and MBP because we found little evidence in favor of the use of oral antibiotics without mechanical bowel preparation. Hoang et al. (23) also implemented a bundle including mechanical preparation and dual antibiotic therapy together, which resulted in a significant decrease in overall SSI. We found striking that Hoang's study included patients undergoing emergency surgeries, in which cases it is difficult to administer mechanical bowel preparation and oral antibiotic therapy.

In our study, besides the decreased infectious complications, there was also a significant decrease in other medical complications such as respiratory failure (8.3%vs.2.2%) and adynamic ileus (32.1%to15.8%). Other studies obtained similar results (25, 28), as opposed to the ERAS® protocols (37), which recommended against mechanical preparation because they considered that it provided no benefits but posed a greater risk of paralytic ileum after surgery.

In addition to trying to reduce SSI with preoperative measures, we included in our bundle some postoperative measures that allowed us for an early diagnosis of severe intra-abdominal infectious complications. After confirming the usefulness of CRP as a biologic marker for the early diagnosis of AL in the Pre-bundle group, we created an algorithm to facilitate the early detection of this complication and proceed accordingly, and to be able to early and safely discharge those patients who had that marker below the pre-established values.

Although we performed CT-scan based mostly on the results of blood tests, the number of them performed was significantly lower than in the Pre-bundle group (29.8% vs. 15.1%); therefore, our measures not only do they decreases the AL rate but allowed for a better selection of patients who required a CT-scan during the postoperative period also. Besides, we succeeded in significantly reducing the number of reoperations from 13.1% to 3.6% (*p* = 0.008), the need of ICU management from 15.5% to 4.3% (*p* = 0.004) and re-hospitalizations from 13.1% to 4.3% (*p* = 0.017), which resulted in a 5-day decrease in hospital stays (11 vs. 6 days *p* = 0.001). These results show that the implementation of a bundle also decrease healthcare costs. Other studies such as the one by Keenan et al. (6) show similar results.

In our study, after conducting the multivariate comparative analysis, we found that the implementation of our bundle proved to be a protective factor from the most important complication in colorectal surgery.

## Limitations

A potential limitation of our study is to be a before-after, single-centre study, rather than a randomized and multicenter one which would provide more reliable results. A larger sample size would have allowed to find more relations between risk factors and complications. Finally, it is a heterogeneous sample, since it encompasses a range of pathologies in colorectal surgery as colorectal cancer, diverticulosis and inflammatory disease, but we wanted to have a clinically representative population sample.

## Conclusions

The implementation of our bundle significantly reduces morbidity adjusted to the severity of complications, the AL rate, hospital stay and re-hospitalizations.

## Data Availability

The datasets presented in this study can be found in online repositories. The names of the repository and accession number can be found below: 10.6084/m9.figshare.17009111&lt.
